# On death and dying – an exploratory and evaluative study of a reflective, interdisciplinary course element in undergraduate anatomy teaching

**DOI:** 10.1186/1472-6920-14-15

**Published:** 2014-01-27

**Authors:** Bernd Alt-Epping, Constanze Lohse, Christoph Viebahn, Nicole von Steinbüchel, Gesine Benze, Friedemann Nauck

**Affiliations:** 1 Department of Palliative Medicine, University Medical Center Göttingen, Göttingen, Germany; 2 Department of Anatomy, University Medical Center Göttingen, Göttingen, Germany; 3 Department of Medical Psychology and Sociology, University Medical Center Göttingen, Göttingen, Germany

## Abstract

**Background:**

Teaching in palliative care aims not only at providing students with specialized knowledge in symptom therapy in advanced disease, but also at developing a professional attitude consistent with the principles and philosophy of palliative care. Reflecting about one’s own or the patient’s death and dying is considered essential for empathic patient care. In medical education the dissection course is often the first encounter with the issue of death and dying and represents a significant emotional challenge to many medical students.

Against this background we implemented a new course element in preparation for the dissection course, offering opportunity to reflect own experiences with death and dying and providing support in finding a balance between authentic empathy and pragmatic action towards deceased persons. We discuss issues such as dignity and professional distance and reason whether guided support for medical students regarding these issues might influence their future attitude as doctors caring for their patients.

**Methods:**

In tandem, we performed a formal evaluation of the seminar and explored the students’ experiences with death and dying, their expectations and fears in the run-up to the dissection course and their attitude towards dissection.

**Results:**

This article describes the structure and the concept of this new interdisciplinary course element and presents the results of the formal course evaluation as well as the explorative part of the accompanying research. Medical students had broad experiences with death and dying even before the dissection course. 89.1% of students had worried about some kind of emotional stress during the dissection course before, but 61.7% stated to have actually perceived emotional stress afterwards. The willingness to donate one's own body for anatomy purposes decreased significantly during the course. The given room for reflection and discussion was appreciated by the students, who felt that the effects of this seminar might be of use even beyond the dissection course.

**Conclusion:**

This new course element successfully assisted medical students during the dissection room experience and gave opportunity to reflection and discussion on death and dying. The accompanying research confirmed the demand for support and gave insight into experiences, emotions and attitudes of medical students.

## Background

Palliative care has been a compulsory subject at the medical faculties in Germany since 2009. Teaching in palliative care aims not only at providing students with specialized knowledge in symptom therapy in advanced disease, but also at developing a professional attitude consistent with the principles and philosophy of palliative care. Reflecting about one’s own or the patient’s death and dying is considered a prerequisite for empathic care, when dealing with severely diseased, dying or other patients in need and requiring complex support [1-3].

In undergraduate teaching, the dissection course is broadly considered to be the first encounter with dead human bodies [4] and therefore offers an appropriate opportunity for discussing matters of death and dying. Moreover, the value of learning anatomy via dissection has been subject to ongoing discussion. An argument against a dissection-based anatomy course is – besides the postulated option to learn anatomy on living people instead of in the dissecting room [5] – the emotional stress which is caused by the handling of deceased people. Many studies have already reported the emotional impact of dissection on medical students [6-10]. In this context, Agnihotri and Sagoo [11] claimed: “We need to prepare the students mentally and emotionally before they enter the dissection room so that they are involved and stimulated”.

We therefore implemented a reflective, interdisciplinary course module at the beginning of the dissection course at the medical faculty in Göttingen, starting in April 2011.

### Course concept and content of the seminar

The course module consists of a preliminary lecture before the students enter the dissecting room for the first time and a seminar (similar to a small-group tutorial) two days later. The lecture addresses the general society’s perception of death and dying, cultural and age-dependent differences and legal and moral implications. The seminar includes topics such as one’s own experiences with illness, dying, death and bereavement, defining criteria of a dignified handling of donated dead bodies in the dissecting room and of deceased patients at their (future) place of work. Furthermore, both lecture and seminar are designed to support the medical students in finding a balance between authentic empathy and pragmatic action related to the contact with the donated bodies and also with respect to later medical activities. In contrast to teacher-centered learning the purpose of the new course module is to encourage discussion and exchange among the students, so that they have the chance to get to know each other better and find a common emotional basis in their learning groups, throughout the complete dissection course.

Students may ask the lecturers for help via email at any time and are provided with the email address of the Department of Palliative Medicine during the lecture and the seminar.

The implementation of this new course module was accompanied by explorative and evaluative research including a formal didactical evaluation and an exploration of the students’ experiences with death and dying, their expectations and fears in the run-up to the dissection course and their attitude towards dissection. In this paper, we will briefly describe the teaching concept and focus on the results of the accompanying research.

## Methods

**Figure 1:**
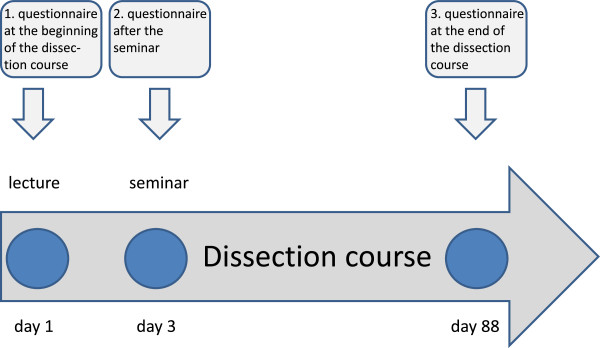
Overview of the dissection course, timing of the new lecture and seminar, and timing of the study questionnaires.

All second-term students enrolled at the medical faculty at the Georg-August-University in Göttingen who participated in the dissection course in the summer term 2011 were addressed. The students were surveyed after the lecture and before entering the dissection room (day 1), during (day 3) and at the end of the dissection course (day 88), using three different questionnaires, developed by the multiprofessional study group (Figure 1). The survey was voluntary and anonymous and was approved by the local cognizant ethics commission.

The first questionnaire included questions about the students’ perceptions and experiences with death and dying, their feelings and fears in the run-up to the dissection course, especially focusing on their concerns about emotional stress, and about stress-induced symptoms and emotional blunting. Finally, the students’ attitudes towards body donation were explored.

The second questionnaire was distributed after the seminar and formally evaluated the seminar, focusing on content, methods and organization.

The third and last questionnaire was distributed at the last day of the dissection course. It included questions about experiences with emotional stress, stress-induced physical symptoms and emotional blunting. Again, the attitudes towards body donation were explored and then compared to the results of the first questionnaire.

Most of the data was collected by using single-choice, multiple-choice and scale questions. For the latter, a 5-point Likert Scale with the items “disagree” (1), “slightly agree” (2), “somewhat agree” (3), “moderately agree” (4) and “strongly agree” (5) was chosen. Some open questions required a qualitative approach, using Mayring’s content analysis [12]. For the answers in the present study which were mostly given in form of bullet points the summary as an analytical technique was applied. Therefore we developed step by step an inductive category system.

The quantitative data was processed with EvaSys, STATISTICA 10, SPSS 19 and Excel 2007. Testing of significance levels, using the Pearson Chi^2^ test and Mann–Whitney-*U*-test, were kept to a minimum due to the exploratory nature of the study, and focused on the pre/post course contrasts.

## Results

From a total of 224 enrolled medical students who signed up for the course, 54.5% returned the first questionnaire, 70.1% the second questionnaire and 67.9% the last one. On average, 37.3% of the participants were male and 62.7% female, with a mean age of 21.9 years (ranged in between 18–33 and in accordance to the general gender distribution of the course).

On first exploration, the medical students stated to have had broad experiences with death and dying: nearly every student (95.8%) had already been involved in the death of a family member or friend; 56.9% had been physically present when a person passed away; 57.5% of the students had attended a severely diseased or dying person over a longer period before the person ultimately died. Before the anatomy course began, most students felt motivated and positive before entering the dissection room; only one fifth (18.9%) had to deal with fearful emotions.

**Figure 2:**
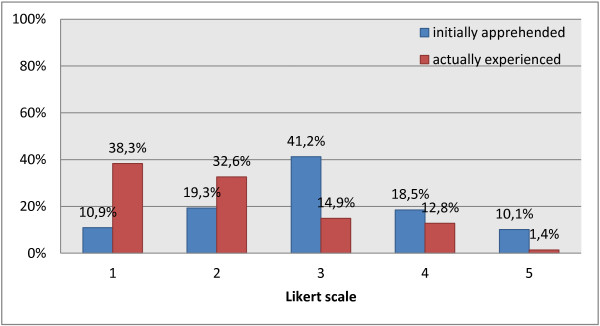
Comparison of initially assumed (first questionnaire, n = 122 out of 224 total) vs. actually experienced (third questionnaire, n = 152 out of 224 total) emotional stress.

Emotional stress, stress-induced symptoms and emotional blunting in connection with the dissection course were determined before and after the course. The results demonstrate that the dissection course triggered emotional stress, even though the experienced stress level was lower than initially assumed by the students themselves. Overall, 61.7% felt some kind of emotional stress during the dissection course, about 89.1% had worried about this previously (p < 0.001, Figure 2). Further results showed that 39.1% of the students suffered from symptoms like fainting, nightmares and loss of appetite in connection with the dissection course. The majority (81.4%) of the respondents agreed to the statement “The dissection room experiences had contributed to a certain emotional blunting”.

Female students were more afraid about the coming dissection course experience than male students (72.7% versus 27.3%) and expected emotional stress more often than men (93.2% versus 80.0%). The aspect of emotional blunting, though, showed no gender specific differences.

More than half of the students had prepared themselves mentally for the anatomy course and specifically for handling of dead human bodies. The respective answers were analyzed qualitatively and condensed on five categories, which are listed according to the frequency they were named.

– C1 apprenticeship/ job

– C2 own experiences

– C3 one’s own initiative

– C4 exchange with social contacts

– C5 religious background

Qualitative analysis revealed a particular emphasis on the first category “apprenticeship/job”. Many respondents were trained as nurses, care attendants or emergency medical assistants before. Traineeship, civilian service and a voluntary year of social service were also allocated to category 1. Some students had experienced cases of death or serious accidents in the family (C2). Self-reflection and literature research were summed up under category 3 “one’s own initiative”. Others felt prepared by relying on social contacts (C4) or because of their religiosity (C5). In total, all respondents considered a special preparation for the anatomy course as necessary.

**Figure 3:**
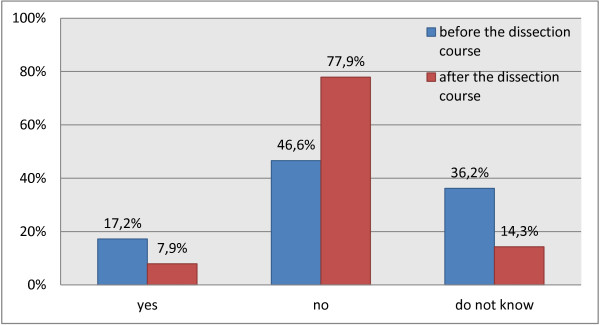
Attitudes of students to the idea of donating their bodies before the dissection course (questionnaire 1, n = 122 out of 224 total) and thereafter (questionnaire 3, n = 152 out of 224 total).

Students were also asked to respond to the idea of donating their body themselves for medical science purposes. Before entering the dissection room, 17.2% of the 122 respondents were in favor of this, but this proportion decreased to 7.9% of 152 respondents at the end of the dissection course (Figure 3). Instead, the number of students who were opposed to the idea of body donation increased from 46.6% (before) to 77.9% (thereafter), but 36.2% (before) and 14.3% (after) of respondents were undecided at that time (Table 1; p < 0.001). So there was a significant difference between the students’ attitude towards body donation before and after the dissection course. Moreover, half of the students directly admitted that their decision was influenced by the dissection course.

**Table 1:** Category system of the question of reasons for the attitude towards body donation

**Yes**	**Don’t know**	**No**
C1 science and teaching	C1 prior agreement with relatives	C1 out of consideration for relatives
C2 acknowledgement of gratitude towards body donors	C2 decision-making after the anatomy course	C2 religiosity
C3 duty of a medical student	C3 not yet able to make a decision	C3 assumption of undignified handling of donated bodies
C4 altruism	C4 yet contemplated this issue	C4 favoring organ donation
		C5 violation of graves

Furthermore, the students were asked about their motives for probable body donation. Most of the students who were in favor of donating their bodies argued with “science and teaching” (C1). The other answers were summed up to the categories “acknowledgement of gratitude towards body donors” (C2), “duty of a medical student” (C3) and “altruism” (C4).

Students who were undecided to donate their own bodies wished to find consent with their families beforehand (C1). Another important reason was their uncertainty what handling of the bodies would effect. Many students desired to await the dissection course and to make their decision thereafter (C2), others felt not able or not yet old enough to make such a decision (C3) or had not yet contemplated this issue.

Students who were decided against donating their own body argued predominantly within the category “out of consideration for relatives” (C1). “Religiosity” (C2), “assumption of undignified handling of the body donors” (C3), “favoring organ donation” (C4) and the “violation of graves” (C5) also played a role.

The formal evaluation of the anatomy course with the additional, new course module was subject of the second questionnaire at the end of the seminar. Overall, the students scored the course with “2” (“good”) on a scale from 1 (“very good”) to 6 (“insufficient”) according to the German school grading system. Formal evaluation showed that students were content with the organization, performance and the applied methods such as working in small-groups.

As part of the formal course evaluation, students appreciated the opportunity for self-reflection. They felt not only stimulated to think about the subject of death and dying, but also felt prepared better for the handling of deceased persons, considering the compiled seminar results on empathy, dignity and distance (93.9% of respondents). The students supported the idea that the effects of the course might be of use also beyond the dissection course.

The last questionnaire included also an overall reflection at the end of the dissection course: nearly all students (99.3%) rated the dissection course as a positive experience, only one student could not agree with that. Nevertheless, 95.9% of the respondents were glad that the dissection course was over.

A large proportion of students (82.1%) agreed to different extents that the dissection course influenced their own clinical attitude; only 17.9% did not agree. A number of respondents “moderately agreed” (39.9%) to the statement that “dissecting became easier with every dissecting experience”.

## Discussion

We introduced a new course element into the dissection course in order to enhance reflection on “death and dying”, being the first contribution to a longitudinal palliative care curriculum. In tandem, we explored the students’ feelings, experiences and attitudes on issues of death and dying, body donation, dissection and their judgment of the new course module. This module intended not only to aid the students through the dissection course, but also to prevent potential negative consequences of a merely technically supervised dissecting course for the students’ future patient-doctor-relationship. By this, we intended to achieve more than just a better preparation for the dissecting room experience, as had been claimed by several authors (see [13]).

The combined evaluative and explorative approach, using a quantitative and qualitative mixed method approach, lead to a comprehensive understanding of students’ perceptions and corresponding, effective teaching intervention.

The high response rates, ranging between 54.5% and 70.1%, and the age and sex distribution allows the conclusion of a representative survey.

The explorative part of this study showed that medical students in Göttingen had had previous experiences with death and dying by far exceeding the percentages described in the literature (34.7% to 81%; [6,7,9,11,14]). Especially the high fatality rate within the families and the large number of students who stated to have witnessed the death of a person might surprise. This might be related to the fact that German medical students are not seldomly involved in the medical field before taking up medical studies (nursing, medical apprenticeships, emergency care) while awaiting approval for a place at university.

Analyses of student responses to their feelings in the run-up to the dissection course showed that most students were motivated and positive before entering the dissecting room only a small proportion had to deal with fear. This is in accordance with other studies, for example by Bernhardt et al. [6] where about 39% of the students were scared of the dissection course but the majority was quite relaxed.

In the present study nearly two thirds had to deal with emotional stress, and 39% even suffered from stress-induced symptoms to a minor degree. Especially the discrepancy between the initially assumed and the actually experienced emotional stress is of note: fewer students had actually suffered from emotional stress, one third did not experience psychological stress at all. This is particularly remarkable as there were individual and varying ways of preparation to the course that were obviously not sufficient to prevent the described high levels of emotional stress. This is also in line with the findings of other studies [6,7,15]. These results indicate that the emotional aspect may be replaced by the immense pressure on learning as the dissection course goes on.

It could be further demonstrated that the attitudes of students towards the idea of becoming body donors themselves changed significantly during the dissection course, so that more than three quarters opposed to the idea at the end of the course. Obviously, this was affected by the dissecting experience. This finding is in consonance with the previous studies by Brinkmann [7] and by Cahill and Ettarh [16].

The formal course evaluation showed that the course element successfully stimulated the students to reflect about the subject of death and dying and that they felt better prepared for the handling of body donors.

Even though the course element was voluntary, the large number of participants was surprising, and all respondents considered a special preparation for the dissection course as necessary. For this reason, this course will be maintained in the future.

There were no needs or responses expressed via the provided email address. Possibly, other options for help seeking should be considered, for example a web forum where students can exchange their experiences among themselves.

This study faces several limitations: due to the voluntary nature of the seminar and the study participation the longitudinal comparability of the data is impaired. This affects for instance the comparability of the students’ attitudes towards body donation or the comparability of assumed or actually experienced emotional stress before and after the dissection course. We did not perform a codification of each questionnaire, so that a respondent could not be followed over all three questionnaires. Therefore, in statistical terms three different student populations were asked, but demographic data showed a homogeneous distribution over all three surveys. Furthermore, long-term outcome of this specific course intervention is not clear and will be subject to future research.

## Conclusions

We demonstrated that there is need for preparing medical students entering the “dissecting room experience”, and that our newly conceptualized course element provided an appropriate, effective and necessary preparation for the dissection course. It stimulated the students to reflect about the topics of death and dying and about their own feelings, and might be of value for their future empathic doctor-patient-relationship, according to their own assessment. Although most students had gathered experiences on “death and dying”, a relevant amount of emotional stress and symptoms in context with the dissection course was detected that require prevention or intervention.

The dissection course contributes to the ritual transformation of students from laypersons to medical practitioners. Traditionally, the body donator can be seen as the first patient. Therefore, this phase during the medical curriculum is particularly vulnerable and requires much attention of all persons involved.

In our university, the palliative care curriculum has taken on this duty, as reflecting death and dying and forming an empathic professional attitude is integral part of palliative care teaching [3]. Nevertheless, this impulse for the anatomy course might well be provided by other departments or faculties.

Our study supports the idea that reflecting on death and dying proves useful for medical students – not only for the dissection course situation.

## Competing interests

The authors declare that they have no competing interests.

## Authors’ contributions

BAE and CL conceived of the concept as well of the study, participated in its design and coordination and drafted the manuscript. CL performed the statistical analysis and drafted the presentation of the results. NvS, CV, GB and FN participated in the design of the course concept and study, its realization into teaching practice, and the critical revision of the manuscript. All authors read and approved the final manuscript.

## Pre-publication history

The pre-publication history for this paper can be accessed here:

http://www.biomedcentral.com/1472-6920/14/15/prepub
